# The Effect of Cranberry Consumption on Body Weight and Liver Enzymes: A Systematic Review and Meta‐Analysis of Randomized Controlled Trials

**DOI:** 10.1002/fsn3.71885

**Published:** 2026-05-12

**Authors:** Sogand Tavakoli, Shadi Ghaemi, Mohammad Reza Amini, Farhang Djafari, Kimia Fathzadeh, Mohammadreza Hosseinpour Dogolsar, Fatemeh Sheikhhossein, Azita Hekmatdoost

**Affiliations:** ^1^ Student Research Committee, Department of Clinical Nutrition & Dietetics, National Nutrition & Food Technology Research Institute Shahid Beheshti University of Medical Sciences Tehran Iran; ^2^ Department of Community Nutrition, School of Nutritional Sciences and Dietetics Tehran University of Medical Sciences (TUMS) Tehran Iran; ^3^ Nutrition and Food Security Research Center Isfahan University of Medical Sciences Isfahan Iran; ^4^ School of Health, Medical and Applied Sciences Central Queensland University Brisbane Australia; ^5^ Nutrition Research Center Tabriz University of Medical Sciences Tabriz Iran; ^6^ Department of Clinical Nutrition, School of Nutritional Sciences and Dietetics Tehran University of Medical Sciences (TUMS) Tehran Iran; ^7^ Department of Clinical Nutrition & Dietetics, National Nutrition & Food Technology Research Institute Shahid Beheshti University of Medical Sciences Tehran Iran

**Keywords:** body weight, cranberry, liver enzymes, randomized controlled trials

## Abstract

This study aimed to review the literature on studies that evaluated the effects of Cranberry consumption on body weight (BW) and liver enzymes in humans. The following databases were searched up to December 2024: PubMed/Medline, Scopus, ISI Web of Science, Embase, Cochrane Library, and Google Scholar. Data were pooled by the use of the inverse variance method and expressed as weighted mean difference (WMD) with 95% confidence intervals (95% CI). Ten studies were included in this meta‐analysis. The results indicated that cranberry consumption significantly reduced body mass index (BMI) (WMD: −0.30 kg/m^2^, 95% CI: −0.55, −0.04, *p* = 0.023) while cranberry did not significantly affect aspartate aminotransferase (AST) (WMD: 1.42 IU/L, 95% CI: −0.80, 3.64, *p* = 0.209), alanine aminotransferase (ALT) (WMD: −0.09 IU/L, 95% CI: −3.63, 3.46, *p* = 0.962), alkaline phosphatase (ALP) (WMD: −0.54 IU/L, 95% CI: −5.83, 4.75, *p* = 0.841), waist circumference (WC) (WMD: −0.37 cm, 95% CI: −1.66, 0.91, *p* = 0.568), and BW (WMD: −0.91 kg, 95% CI: −2.09, 0.27, *p* = 0.132). Cranberry consumption did lead to a reduction in BMI among adults over 50 years, those classified as overweight, and participants treated for more than 8 weeks. Conversely, cranberry intake was also linked to a significant increase in AST levels within these subgroups. Overall, the findings suggest that cranberry supplementation notably reduced BMI, particularly among older adults, overweight individuals, and participants who engaged in the intervention for more than 8 weeks.

## Graphical abstract

Cranberry consumption significantly reduced BMI but had no meaningful effects on body weight, waist circumference, or liver enzymes. The BMI‐lowering effect was more pronounced in older adults, overweight individuals, and interventions lasting longer than 8 weeks.

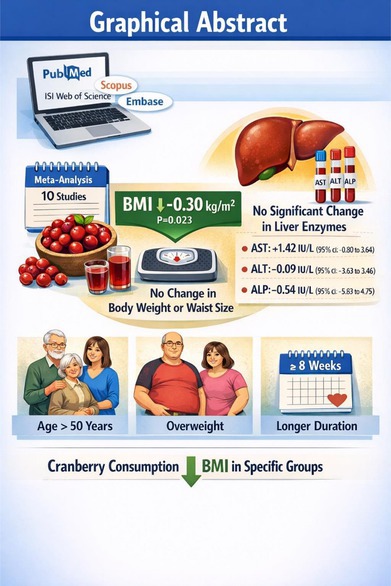

## 1 Introduction

Recently, chronic diseases such as liver dysfunction, cancer, diabetes, and cardiovascular diseases (CVDs) have become the leading causes of mortality globally (Dugani and Gaziano 2016). Modifiable risk factors, including alcohol consumption, high body mass index (BMI), cigarette smoking, unhealthy diet, and physical inactivity play crucial roles in the development of chronic diseases (Kearns et al. 2014; Ng et al. 2020). Overweight and obesity, defined as exceeding the normal range of BMI or waist circumference (WC) along with other anthropometric indices, can lead to chronic diseases and negatively impact health (Sweatt et al. 2024). Evidence indicates that in the 21st century, obesity has affected over 600 million adults worldwide, and as its prevalence increases, a rise in associated disorders is anticipated (Cibičková et al. 2019; GBD 2017 Disease and Injury Incidence and Prevalence Collaborators 2018; Kearns et al. 2014). Additionally, obesity can contribute to liver diseases and abnormalities in liver enzymes levels, including aspartate aminotransferase (AST), alanine aminotransferase (ALT), and alkaline phosphatase (ALP) (Aragon and Younossi 2010). ALT and AST are key enzymes found in various organs; elevated levels of them indicate hepatocellular injury (Kalas et al. 2021). In cases of bile duct epithelium obstruction, ALP plays a crucial role and reflects liver dysfunction (Aragon and Younossi 2010; Kalas et al. 2021; Qin et al. 2023). Well‐established evidence demonstrates that high liver enzymes levels are linked to hard endpoints in liver disease, such as mortality and the need for liver transplantation (Lee, Kim, et al. 2008; Lee et al. 2012). Some research has suggested that lifestyle modifications, including dietary changes and weight management, are fundamental steps for improving liver health and managing metabolic conditions (Abenavoli et al. 2017; Eslamparast et al. 2014; Faghihzadeh et al. 2014).

Fruits and vegetables, particularly berries, are known as sources of bioactive compounds and are emphasized for their crucial role in lowering the risk of metabolic disorders and reducing inflammatory markers (Ruel and Couillard 2007; Salomone et al. 2016; Slavin and Lloyd 2012). Cranberries ( *Vaccinium macrocarpon* ) are distinguished by their antioxidant properties and unique polyphenol profile, notably their high content of A‐type proanthocyanidins, which are rare in most other fruits (Blumberg et al. 2013; Skrovankova et al. 2015). Numerous animal studies have concluded that cranberries positively impact liver enzyme levels, oxidative stress, and hepatic inflammation (Faheem et al. 2020; Glisan et al. 2016; Valenti et al. 2013).

A study by Hormoznejad et al. found significant changes in ALT levels between the cranberry and placebo groups, though no changes were observed in AST and ALP levels (Hormoznejad et al. 2020). A systematic review and meta‐analysis has revealed that cranberry supplementation significantly reduces BMI but has no effect on WC (Pourmasoumi et al. 2020). In contrast, another meta‐analysis indicated that cranberry consumption was associated with a reduction in waist circumference (Hormoznejad et al. 2021). Moreover, the last two systematic reviews and meta‐analyses in this context focused on the impact of cranberry supplementation on glycemic and lipid profiles, as well as blood pressure (Delpino et al. 2024; Li et al. 2024). Other systematic reviews have also investigated the effects of cranberry consumption on CVD risk factors, including BMI and WC (Hormoznejad et al. 2021; Pourmasoumi et al. 2020). Therefore, none of these reviews have examined the effect of cranberry intake on liver enzymes. Consequently, considering the uncertainties and conflicting findings of previous research, along with the lack of comprehensive analysis to investigate the effects of cranberry intake on body weight (BW) and liver enzymes, this study aimed to systematically review and meta‐analyze the impact of cranberry consumption on these outcomes.

## 2 Materials & Methods

The current systematic review and meta‐analysis was performed in accordance with the Preferred Reporting Items for Systematic Reviews and Meta‐analysis (PRISMA) guidelines (Moher et al. 2015). This study's registration number in PROSPERO is CRD420251028540.

### 2.1 Search Strategy

Different medical databases including PubMed/Medline, Scopus, ISI Web of Science, Embase, Cochrane Library, and Google Scholar were systematically searched from their inception until December 2024. Two independent researchers utilized the following keywords such as (cranberry[tiab] OR “ *Vaccinium macrocarpon* ”[tiab] OR “ *Vaccinium microcarpum* ”[tiab] OR “ *Vaccinium oxycoccus* ”[tiab] OR “ *Vaccinium erythrocarpum* ”[tiab] OR “ *Vaccinium macrocarpon* ”[Mesh]) AND (“Body Weight”[tiab] OR “Quetelet Index”[tiab] OR “Body Mass Index”[tiab] OR “Body Weight”[Mesh] OR “Body Weight Changes”[Mesh] OR “Body Mass Index”[Mesh] OR “Weight Loss”[Mesh] OR Obesity[Mesh] OR “Waist Circumference”[Mesh] OR “Obesity, Abdominal”[Mesh] OR “Fasting Blood Sugar”[tiab] OR “Blood Glucose”[Mesh] OR “alanine aminotransferase” [Mesh] OR “alanine aminotransferase”[tiab] OR ALT[tiab] OR “aspartate aminotransferases”[Mesh] OR “aspartate aminotransferases”[tiab] OR AST[tiab] OR “alkaline phosphatase”[Mesh] OR “alkaline phosphatase”[tiab] OR ALP[tiab]) for search. To identify additional studies, forward and backward reference searches were performed on the included articles. Unpublished manuscripts, conference papers, and theses were not considered in this study.

### 2.2 Eligibility Criteria

The PICOS model (Richardson et al. 1995) was followed to establish the eligibility criteria as follows: population (age > 18 years), intervention (Cranberry consumption), comparator (placebo), outcome (BW, BMI, WC, ALT, AST, and ALP) and study design (parallel and cross‐over clinical trials). To be included in this meta‐analysis, studies must meet the following criteria: (a) randomized clinical trials (RCTs) comparing the effect of Cranberry consumption to placebo; (b) included adult participants 18 years of age and older; (c) reported results as mean and standard deviation (SD) for BW, BMI, WC, ALT, AST, and ALP; and (d) published in English.

Studies were excluded if they met any of the following criteria: (a) non‐RCTs studies; (b) performed on animals; (c) involved individuals < 18 years old, (d) studies with no placebo group; (e) examined the effect of other interventions along with Cranberry consumption; and (f) did not report outcome (BW, BMI, WC, ALT, AST, and ALP) at baseline and at the end of the intervention. Additionally, books, letters, comments, conference papers, and review articles were excluded.

### 2.3 Data Extraction

Two researchers (ST and MRA) independently extracted data for this meta‐analysis, performed the quality assessment of the included studies, and cross‐verified the results. Any disagreements were resolved by a third independent investigator (AH). Data extraction followed a standardized format for both the intervention and control groups and included study characteristics (authors, publication year, study design, country, trial duration, and intervention arms), participant details (inclusion criteria, age, sex, and health status), and assessed outcomes (baseline and final values for BW, BMI, WC, ALT, AST, and ALP). For the outcome values, the mean and SD changes were extracted. If a study did not report these changes, the baseline and final values were used for both the intervention and control groups. When additional details were needed, the study authors were contacted.

### 2.4 Quality Assessment

The Revised Cochrane risk‐of‐bias tool (RoB 1) (Higgins et al. 2019) was utilized to evaluate the quality of included studies by two researchers (ST and MRA). The RoB 1 assesses randomized trials via different domains as follows: random sequence generation, allocation concealment, blinding of participants and personnel, blinding of outcome assessment, incomplete outcome data, selective reporting, and other potential sources of bias. Based on the Cochrane Handbook guidelines, studies were classified as having a low, high, or unclear risk of bias for each domain.

### 2.5 Data Synthesis and Statistical Analysis

The effect of Cranberry on BW, BMI, WC, ALT, AST, and ALP was estimated by pooling baseline and final mean and SD values of the studies in both intervention and control groups. The formula SD^2^ baseline + SD^2^ final − (2 R * SD baseline + SD final) (Borenstein et al. 2009) was used to calculate the SD of the mean difference for studies that did not report, where R was considered to be 0.8. Weighted mean differences (WMDs) with 95% confidence intervals (CIs) were calculated to assess the size and direction of the effect, with negative values indicating a decrease in these measures. The 95% CI provides an estimate of precision, where narrower intervals represent more dependable results. The heterogeneity was evaluated using the I‐squared (I^2^) statistic, with substantial heterogeneity considered as *I* ^2^ > 50% and a *p*‐value < 0.1 based on Cochrane's test. The Der Simonian and Laird random‐effects model was employed. A subgroup analysis was performed to explore potential differences in the effects of Cranberry consumption on BW, BMI, and WC according to age, BMI categories, duration, and type of intervention. In addition, potential differences in the effects of Cranberry consumption on ALT and AST were analyzed based on age, BMI categories, intervention duration, type of intervention, and sex. The fixed‐effects model was used to calculate the pooled effect sizes within each subgroup. Additionally, heterogeneity was assessed using the *I* ^2^ statistic and *p*‐values for subgroup differences to evaluate variability within and between groups. Sensitivity analysis was performed to explore the effect of each study on overall analysis. Furthermore, publication bias was evaluated using Egger's regression test (Egger et al. 1997). The meta‐analysis was performed using STATA software, version 14 (Stata Corp LP, College Station, TX, USA).

## 3 Results

### 3.1 Study Selection

**FIGURE 1:**
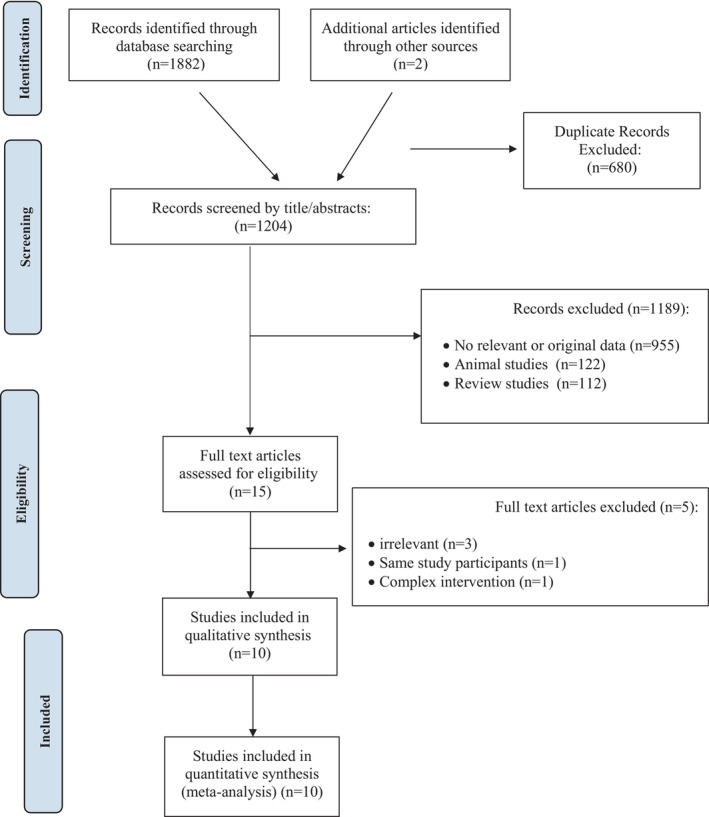
Flow chart of the number of studies identified and selected into the meta‐analysis.

The initial search resulted in 1884 articles (Figure 1). After removing duplicates, 1204 articles remained for screening based on title and abstract. A total of 1189 articles were excluded, leaving 15 papers for full‐text review. Following a thorough evaluation, 5 articles were excluded for the following reasons: (a) irrelevant data (*n* = 3), (b) same study participants (*n* = 1) (Rosa et al. 2023), and (c) complex intervention (*n* = 1) (Rahn et al. 2023). Finally, 10 articles were included in this meta‐analysis.

### 3.2 Study Characteristics

The characteristics of the final studies are shown in (Table 1). The sample size varied between 19 (Zare Javid et al. 2018) to 94 (Masnadi Shirazi et al. 2021). The participants' mean age ranged between 21.6 (Valentova et al. 2007) to 65.5 (Flanagan et al. 2022; Lee, Chan, et al. 2008) years old. The studies were conducted in Iran (Eftekhari et al. 2016; Hormoznejad et al. 2020; Masnadi Shirazi et al. 2021; Zare Javid et al. 2018), Brazil (de Souza Gouveia Moreira et al. 2024; Fatel et al. 2021), USA (Hsia et al. 2020), UK (Flanagan et al. 2022), Taiwan (Lee, Chan, et al. 2008), and Czech Republic (Valentova et al. 2007). The mean of the baseline BMI for participants was 28.7 kg/m^2^. Studies involved healthy participants (Flanagan et al. 2022; Valentova et al. 2007), non‐alcoholic fatty liver disease (NAFLD) (Hormoznejad et al. 2020; Masnadi Shirazi et al. 2021), patients with type 2 diabetes (Lee, Chan, et al. 2008; Zare Javid et al. 2018), elevated fasting glucose or impaired glucose tolerance (Hsia et al. 2020), metabolic syndrome (Eftekhari et al. 2016), rheumatoid arthritis (Fatel et al. 2021), and chronic kidney disease (stages 3–4) (de Souza Gouveia Moreira et al. 2024). Studies published from 2007 to 2024.

**TABLE 1:** Demographic characteristics of the included studies.

First author (year)	Location	Study design	Health status	Sex	Sample size	Duration (week)	Mean age (year)	Baseline BMI (kg/m^2^)	Intervention group	Comparator group	Outcome
1. Eftekhari et al. (2016)	Iran	RCT, parallel	Metabolic syndrome	Female	48	8	42	29.3	400 mg cranberry supplement	Placebo	WC
2. Fatel et al. (2021)	Brazil	RCT, parallel	Rheumatoid arthritis	Both	41	12	58	26.7	500 mL reduced‐calorie cranberry juice and 3 g of fish oil n‐3 fatty acids	3 g of fish oil n‐3 fatty acids	Weight/BMI/WC
3. Flanagan et al. (2022)	United Kingdom	RCT, parallel	Healthy	Both	60	12	65.5	25	9 g freeze‐dried cranberry powder or 100 g of fresh cranberries	Placebo	Weight/BMI/ALT/AST/ALP
4. de Souza Gouveia Moreira et al. (2024)	Brazil	RCT, parallel	Chronic kidney disease (Stages 3–4)	Both	25	8	57.7	29.7	1 g cranberry extract	Placebo	BMI
5. Hormoznejad et al. (2020)	Iran	RCT, parallel	NAFLD	Both	41	12	41.7	32	288 mg cranberry supplementation (equal to 26 g dried cranberry fruit) + weight loss diet	Placebo + weight loss diet	Weight/BMI/WC/ALT/AST/ALP
6. Hsia et al. (2020)	USA	RCT, parallel	Elevated fasting glucose or impaired glucose tolerance	Both	35	8	47.5	36.9	450 mL cranberry juice	Placebo	Weight
7. Lee, Chan, et al. (2008)	Taiwan	RCT, parallel	Type 2 diabetes	Both	30	12	65.5	26	500 mg cranberry extract	Placebo	BMI/WC/ALT/AST
8. Masnadi Shirazi et al. (2021)	Iran	RCT, parallel	NAFLD	Both	94	24	43.1	28.4	144 mg *Vaccinium macrocarpon* (equal to 13 g dried cranberry fruit)	Placebo	BMI/ALT/AST/ALP
9. Valentova et al. (2007)	Czech Republic	RCT, parallel	Healthy	Female	65	8	21.6	21	400 and 1200 mL dried cranberry juice	Placebo	ALT/AST
10. Zare Javid et al. (2018)	Iran	RCT, parallel	Type 2 Patients with Diabetes with Periodontal Disease	Both	19	8	55.4	27.7	400 ml cranberry juice +2 g omega‐3	2 g omega‐3	Weight/BMI/WC

Abbreviations: ALP, alkaline phosphatase; ALT, alanine aminotransferase; AST, aspartate aminotransferase; BMI, body mass index; NAFLD, non‐alcoholic fatty liver disease; RCT, randomized controlled trial; WC, waist circumference.

The intervention duration was from 8 to 24 weeks (de Souza Gouveia Moreira et al. 2024; Eftekhari et al. 2016; Hsia et al. 2020; Valentova et al. 2007; Zare Javid et al. 2018) (Masnadi Shirazi et al. 2021). All studies employed a parallel design. Various types and dosage of Cranberry were used in the intervention group, including Cranberry supplements (400 and 288 mg), Cranberry juice (1200, 500, 450, and 400 mL), and Cranberry extract (1 g, 500 mg, and 144 mg). Notably, in two studies, the intervention group received cranberry juice plus 3 g (Fatel et al. 2021) and 2 g (Zare Javid et al. 2018) of fish oil n‐3 fatty acids. Consequently, in these studies, the control group also received 3 and 2 g of fish oil n‐3 fatty acids, respectively. In one study (Hormoznejad et al. 2020), the intervention group followed a weight lost dietary plan in addition to Cranberry supplementation while the control group followed the same weight‐loss dietary plan but received a placebo instead of Cranberry. In the remaining seven studies, the control group received a placebo.

Out of the included studies, five (Fatel et al. 2021; Flanagan et al. 2022; Hormoznejad et al. 2020; Hsia et al. 2020; Zare Javid et al. 2018) reported BW, seven (de Souza Gouveia Moreira et al. 2024; Fatel et al. 2021; Flanagan et al. 2022; Hormoznejad et al. 2020; Lee, Chan, et al. 2008; Masnadi Shirazi et al. 2021; Zare Javid et al. 2018) examined BMI, five (Eftekhari et al. 2016; Fatel et al. 2021; Hormoznejad et al. 2020; Lee, Chan, et al. 2008; Zare Javid et al. 2018) assessed WC, five (Flanagan et al. 2022; Hormoznejad et al. 2020; Lee, Chan, et al. 2008; Masnadi Shirazi et al. 2021; Valentova et al. 2007) reported ALT and AST, and three (Flanagan et al. 2022; Hormoznejad et al. 2020; Masnadi Shirazi et al. 2021) investigated ALP as an outcome.

After quality assessment of studies, six studies were not fully clear about their methodology for allocation concealment (de Souza Gouveia Moreira et al. 2024; Fatel et al. 2021; Flanagan et al. 2022; Lee, Chan, et al. 2008; Valentova et al. 2007; Zare Javid et al. 2018). Two studies showed high risk of bias for blinding the participants and personnel and outcome assessment (Fatel et al. 2021; Zare Javid et al. 2018). In addition, seven studies demonstrated unknown risk of bias for blinding the outcome assessment (de Souza Gouveia Moreira et al. 2024; Eftekhari et al. 2016; Flanagan et al. 2022; Hormoznejad et al. 2020; Hsia et al. 2020; Lee, Chan, et al. 2008; Valentova et al. 2007). Moreover, two studies were identified for having high risk of bias for other sources (de Souza Gouveia Moreira et al. 2024; Zare Javid et al. 2018) (Table 2).

**TABLE 2:** Risk of bias for randomized controlled trials, assessed according to the Revised Cochrane risk‐of‐bias tool for randomized trials.

Publications	Random sequence generation	Allocation concealment	Selective reporting	Blinding (participants and personnel)	Blinding (outcome assessment)	Incomplete outcome data	Other source of bias
1. Eftekhari et al. (2016)	L	L	L	L	U	L	L
2. Fatel et al. (2021)	L	U	L	H	H	L	L
3. Flanagan et al. (2022)	L	U	L	L	U	L	L
4. de Souza Gouveia Moreira et al. (2024)	L	U	L	L	U	L	H
5. Hormoznejad et al. (2020)	L	L	L	L	U	L	L
6. Hsia et al. (2020)	L	L	L	L	U	L	L
7. Lee, Chan, et al. (2008)	L	U	L	L	U	L	L
8. Masnadi Shirazi et al. (2021)	L	L	L	L	L	L	L
9. Valentova et al. (2007)	L	U	L	L	U	L	L
10. Zare Javid et al. (2018)	L	U	L	H	H	L	H

Abbreviations: H, high risk of bias; L, low risk of bias; U, unknown.

### 3.3 Effect of Cranberry Consumption on BW

**FIGURE 2:**
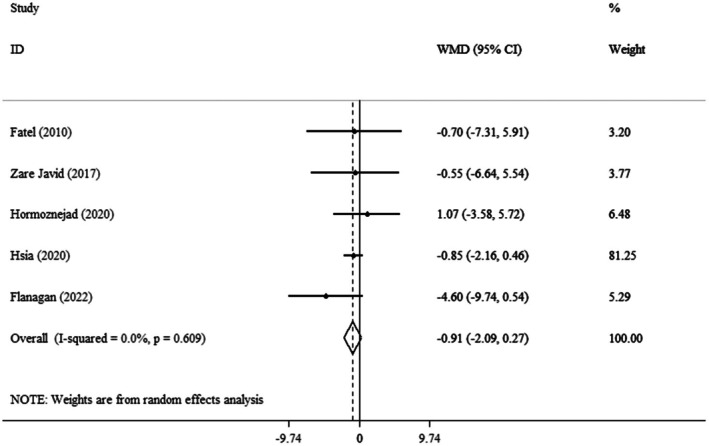
Forest plot detailing weighted mean difference and 95% confidence intervals (CIs) for the effect of cranberry consumption on weight.

The random effect model illustrated that the pooled mean effect size was not significant (WMD: −0.91 kg, 95% CI: −2.09, 0.27, *p* = 0.132) (*I* ^2^ = 0.0%, *p* = 0.609) compared to the control group (Figure 2). Also, subgroup analysis revealed no significant differences in BW changes with Cranberry consumption across age groups, BMI categories, type of intervention, or intervention durations (Table 3).

**TABLE 3:** Subgroup analysis of included randomized controlled trials in meta‐analysis of the effect of cranberry consumption on body weight and liver enzymes.

Group	No. of trials	WMD (95% CI)	*p* value	*I* ^2^ (%)	P‐heterogeneity	P for between subgroup heterogeneity
Weight						
Type of intervention						
Powder	2	−1.48 (−4.93, 1.97)	0.40	61.1	0.10	0.72
Juice	3	−0.83 (−2.09, 0.43)	0.19	0.0	0.99	
Duration (week)						
≤ 8	2	−0.84 (−2.12, 0.45)	0.20	0.0	0.92	0.77
> 8	3	−1.31 (−4.37, 1.74)	0.40	23.5	0.27	
Age						
≤ 50	2	−0.71 (−1.97, 0.55)	0.27	0.0	0.43	0.37
> 50	3	−2.34 (−5.71, 1.04)	0.17	0.0	0.51	
Mean BMI						
25–29.9	3	−2.34 (−5.71, 1.04)	0.17	0.0	0.51	0.37
≥ 30	2	−0.71 (−1.97, 0.55)	0.27	0.0	0.43	
BMI						
Type of intervention						
Powder	5	−0.29 (−0.55, −0.04)	0.02	0.0	0.45	0.89
Juice	2	−0.41 (−2.05, 1.24)	0.62	0.0	0.85	
Duration (week)						
≤ 8	2	−0.07 (−2.13, 1.99)	0.94	0.0	0.94	0.72
> 8	5	−0.30 (−0.56, −0.04)	0.02	0.0	0.46	
Age						
≤ 50	2	0.29 (−0.74, 1.31)	0.58	0.0	0.92	0.24
> 50	5	−0.33 (−0.60, −0.07)	0.01	0.0	0.67	
Mean BMI						
25–29.9	6	−0.31 (−0.56, −0.05)	0.01	0.0	0.92	0.52
≥ 30	1	0.38 (−1.70, 2.46)	0.72	—	—	
WC						
Type of intervention						
Powder	3	−0.37 (−1.73, 10.99)	0.59	0.0	0.53	0.99
Juice	2	−0.40 (−4.19, 3.40)	0.83	0.0	0.72	
Duration (week)						
≤ 8	2	−0.73 (−2.98, 1.53)	0.52	0.0	0.67	0.70
> 8	3	−0.20 (−1.76, 1.36)	0.79	0.0	0.58	
Age						
≤ 50	2	−0.20 (−2.28, 1.88)	0.85	18.2	0.26	0.83
> 50	3	−0.48 (−2.11, 1.15)	0.56	0.0	0.93	
Mean BMI						
25–29.9	4	−0.62 (−1.98, 0.74)	0.37	0.0	0.97	0.27
≥ 30	2	1.68 (−2.25, 5.61)	0.40	—	—	
ALT						
Type of intervention						
Powder	4	0.86 (−1.16, 2.88)	0.40	76	0.005	0.62
Juice	1	−0.03 (−2.99, 2.93)	0.98	—	—	
Duration (week)						
≤ 8	1	−0.03 (−2.99, 2.93)	0.98	—	—	0.62
> 8	4	0.86 (−1.16, 2.88)	0.40	76	0.005	
Age						
≤ 50	3	0.83 (−1.52, 3.18)	0.48	84.1	0.002	0.76
< 50	2	0.32 (−2.06, 2.69)	0.79	0.0	0.59	
Mean BMI						
< 25	1	−0.03 (−2.99, 2.93)	0.98	—	—	0.01
25–29.9	3	1.55 (−0.53, 3.63)	0.14	57.5	0.09	
≥ 30	1	−11.5 (−20.3, −2.70)	0.01	—	—	
Sex						
Both	4	0.86 (−1.16, 2.88)	0.40	76.4	0.005	0.62
Female	1	−0.03 (−2.99, 2.93)	0.98	—	—	
AST						
Type of intervention						
Powder	4	2.49 (1.00, 3.99)	0.001	8.80	0.34	0.009
Juice	1	−1.06 (−3.24, 1.13)	0.34	—	—	
Duration (week)						
≤ 8	1	−1.06 (−3.24, 1.13)	0.34	—	—	0.009
> 8	4	2.49 (1.00, 3.99)	0.001	8.80	0.34	
Age						
≤ 50	3	0.47 (−1.30, 2.24)	0.60	72.1	0.02	0.16
> 50	2	2.20 (0.48, 3.92)	0.01	12.7	0.28	
Mean BMI						
< 25	1	−1.06 (−3.24, 1.13)	0.34	—	—	0.009
25–29.9	4	2.49 (1.00, 3.99)	0.001	8.80	0.34	
Sex						
Both	4	2.49 (1.00, 3.99)	0.001	8.80	0.34	0.009
Female	1	−1.06 (−3.24, 1.13)	0.34	—	—	

Abbreviations: ALT, alanine aminotransferase; AST, aspartate aminotransferase; BMI, body mass index; WC, waist circumference; WMD, weight mean difference.

### 3.4 Effect of Cranberry Consumption on BMI

**FIGURE 3:**
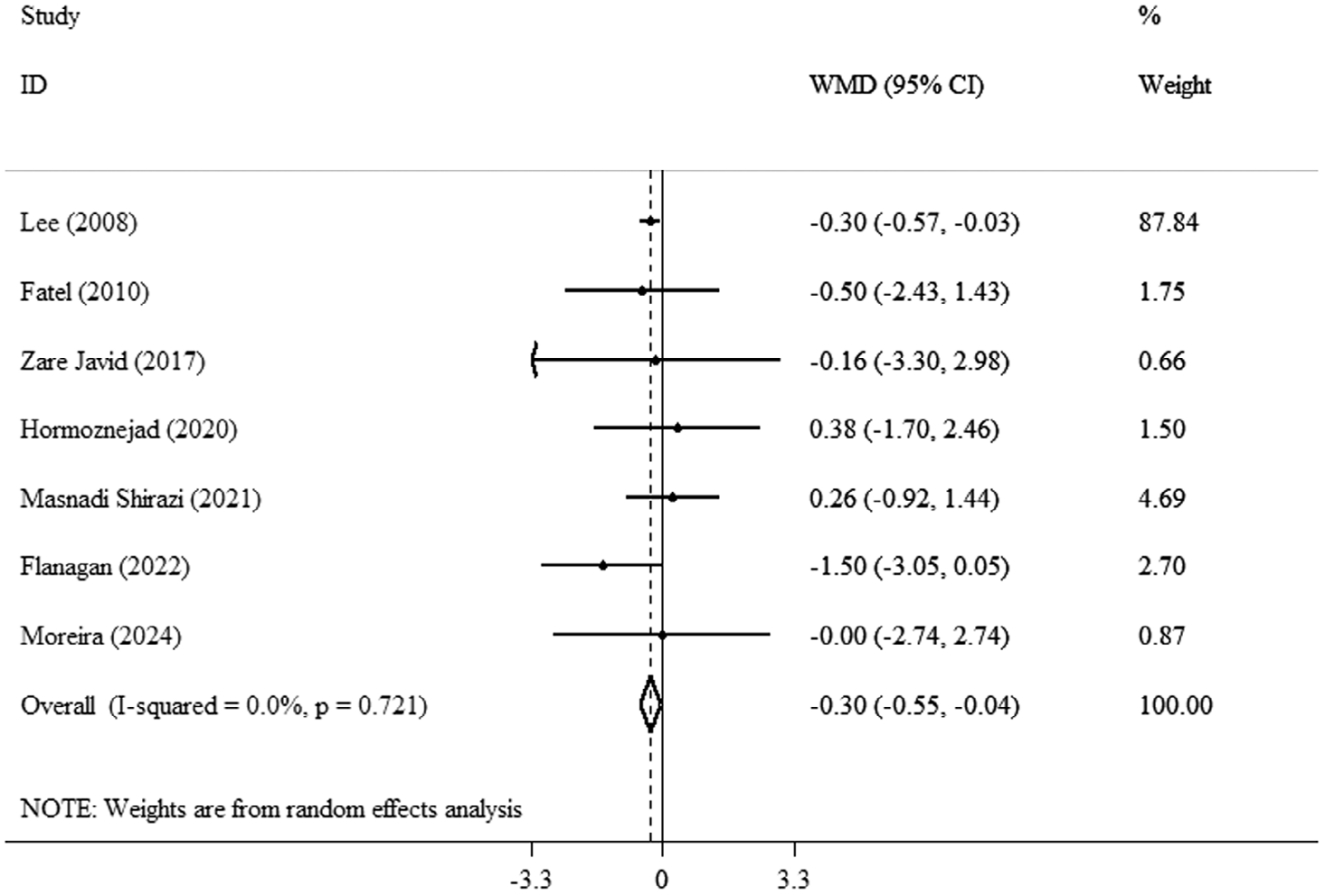
Forest plot detailing weighted mean difference and 95% confidence intervals (CIs) for the effect of cranberry consumption on BMI.

The pooled mean effect size was significant (WMD: −0.30 kg/m^2^, 95% CI: −0.55, −0.04, *p* = 0.023) (*I* ^2^ = 0.0%, *p* = 0.721) compared to the control group (Figure 3). Findings from the subgroup analysis showed that the consumption of cranberry in powdered form for more than 8 weeks, among individuals over the age of 50 and those classified as overweight based on BMI, significantly reduced BMI (Table 3).

### 3.5 Effect of Cranberry Consumption on WC

**FIGURE 4:**
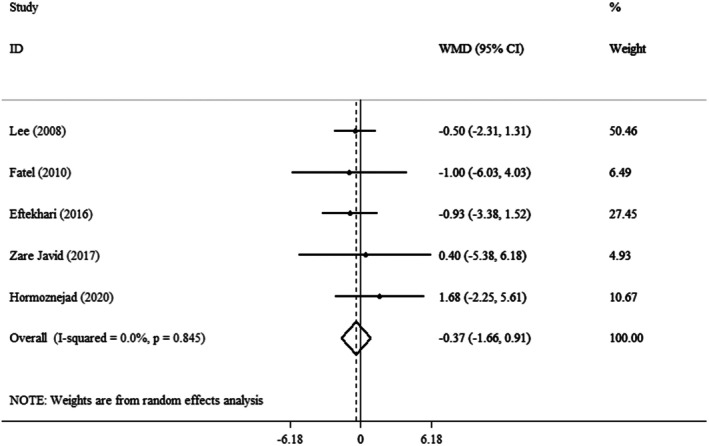
Forest plot detailing weighted mean difference and 95% confidence intervals (CIs) for the effect of cranberry consumption on WC.

This meta‐analysis found that the pooled mean effect size was not significant (WMD = −0.37 cm, 95% CI: −1.66, 0.91, *p* = 0.568) (*I* ^2^ = 0.0%, *p* = 0.845) compared to the control group (Figure 4). Additionally, the subgroup analysis showed no significant variation in WC changes associated with Cranberry consumption across different age groups, BMI categories, intervention types, or durations (Table 3).

### 3.6 Effect of Cranberry Consumption on ALT

**FIGURE 5:**
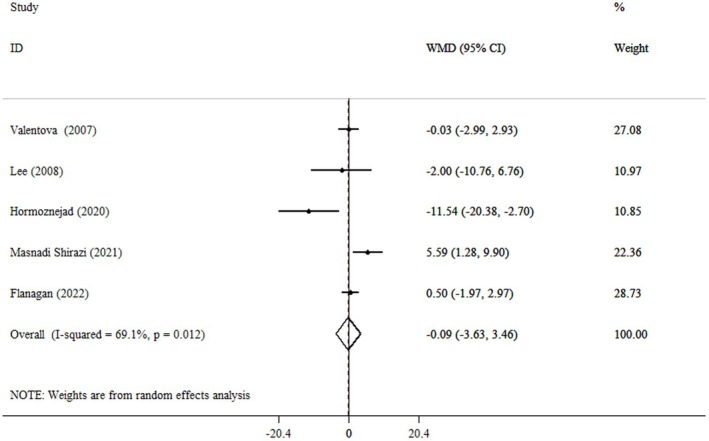
Forest plot detailing weighted mean difference and 95% confidence intervals (CIs) for the effect of cranberry consumption on ALT.

Based on the pooled analysis, the effect of Cranberry consumption on ALT was not significant (WMD = −0.09 IU/L, 95% CI: −3.63, 3.46, *p* = 0.962) (*I* ^2^ = 69.1%, *p* = 0.012) compared to the control group (Figure 5). Based on the results from subgroup analysis, the cranberry consumption significantly decreased the ALT levels of those categorized as obese based on BMI. The consumption of Cranberry did not significantly change the ALT levels across different age groups, intervention types, or durations (Table 3).

### 3.7 Effect of Cranberry Consumption on AST

**FIGURE 6:**
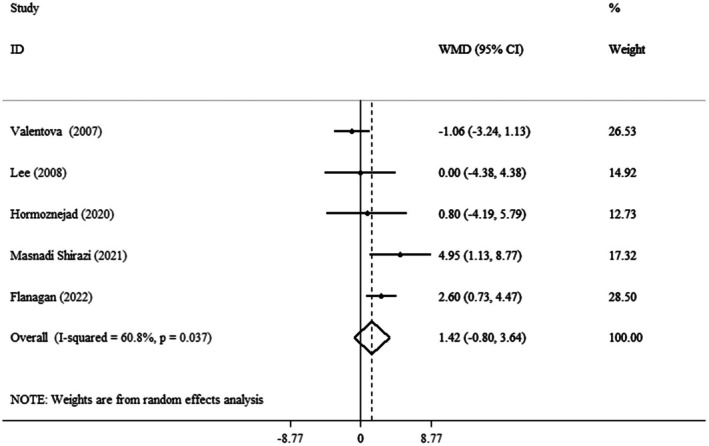
Forest plot detailing weighted mean difference and 95% confidence intervals (CIs) for the effect of cranberry consumption on AST.

The pooled mean effect size was not significant (WMD = 1.42 IU/L, 95% CI: −0.80, 3.64, *p* = 0.209) (*I* ^2^ = 60.8%, *p* = 0.037) compared to the control group (Figure 6). Subgroup analysis revealed that the powdered form of the cranberry consumption for more than 8 weeks, among individuals over the age of 50, those classified as overweight based on BMI, and both sexes, significantly increased AST levels (Table 3).

### 3.8 Effect of Cranberry Consumption on ALP

**FIGURE 7:**
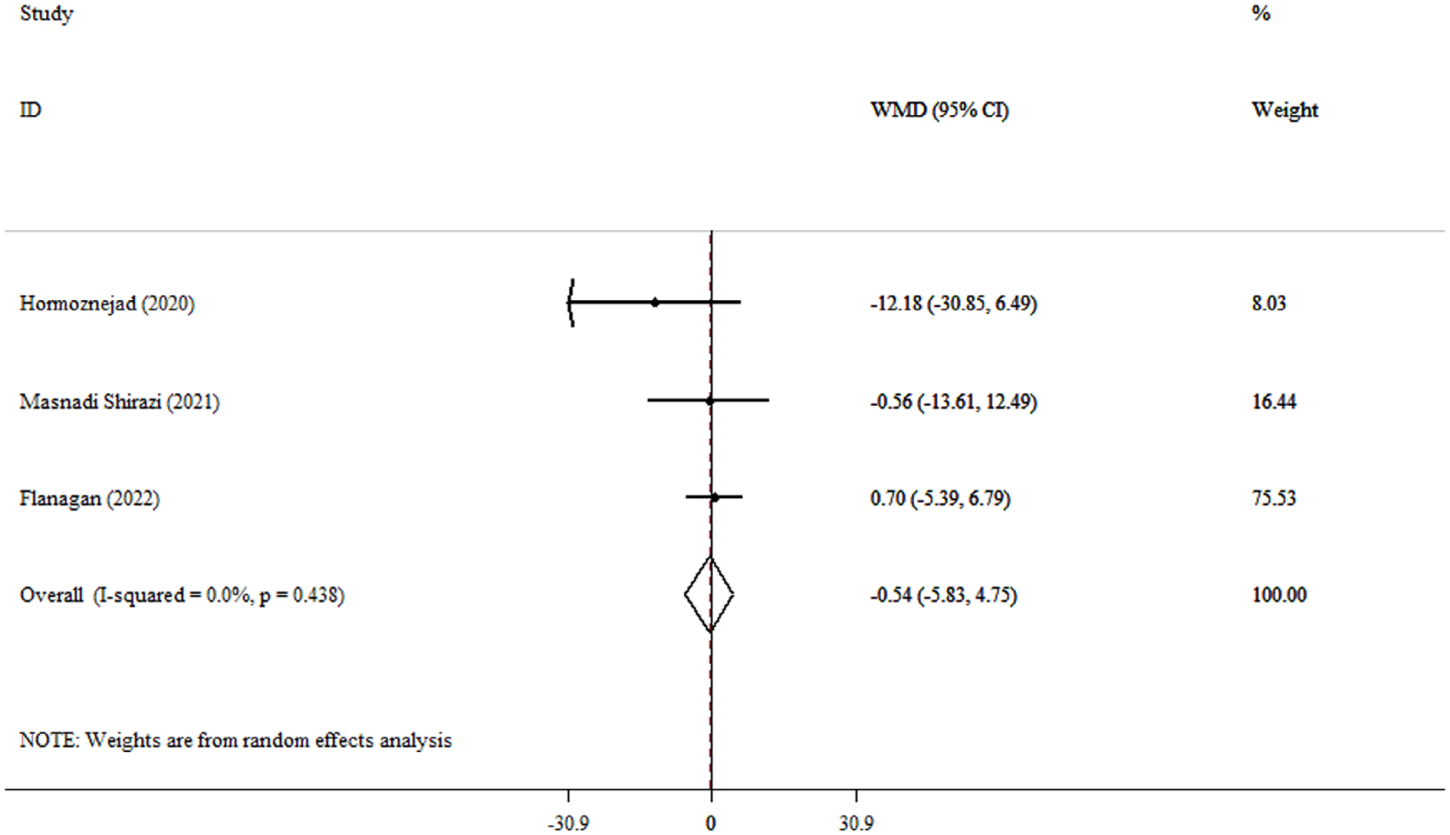
Forest plot detailing weighted mean difference and 95% confidence intervals (CIs) for the effect of cranberry consumption on ALP.

The current meta‐analysis showed that the pooled mean effect size was not significant (WMD = −0.54 IU/L, 95% CI: −5.83, 4.75, *p* = 0.841) (*I* ^2^ = 0.0%, *p* = 0.438) compared to the control group (Figure 7).

### 3.9 Sensitivity Analysis

To evaluate the impact of each study on the overall pooled effect size, studies were systematically excluded one at a time from the analysis. The sensitivity analysis revealed that removing any individual study did not change the overall estimates.

### 3.10 Publication Bias

Egger's weighted regression tests was conducted to explore the publication bias. The results of Egger's test demonstrated no publication bias for BW (*p* = 0.827), BMI (*p* = 0.955), WC (*p* = 0.477), ALT (*p* = 0.494), AST (*p* = 0.933), and ALP (*p* = 0.356).

## 4 Discussion

Despite growing interest in cranberry supplementation for metabolic health, its effects on anthropometric measures and liver enzymes have not been systematically evaluated. Herein, we conducted this systematic review and meta‐analysis to comprehensively investigate the impact of cranberry supplementation on BW and liver enzyme levels in adults. Based on our findings, cranberry consumption led to a statistically significant reduction in BMI, although no significant effects were observed for BW, WC, ALT, AST, or ALP in the overall analysis. Subgroup analyses indicated that cranberry powder consumption significantly reduced BMI among adults aged over 50 years, those classified as overweight, and participants treated for longer than 8 weeks. However, cranberry powder intake was also associated with a significant increase in AST levels within these subgroups.

In line with our findings, Pourmasoumi et al. reported that cranberry supplementation led to a significant reduction in systolic blood pressure and BMI but had no effect on WC, lipid profiles, glycemic and inflammatory markers (Pourmasoumi et al. 2020). While we did not observe any significant changes in liver enzymes (ALT, AST, and ALP), their study similarly reported no effect on lipid profiles, despite the theoretical expectation that cranberry polyphenols might favorably influence lipid metabolism. The parallel absence of effects on lipid profiles and liver enzymes across both studies suggests that differences in the baseline health status of participants, including variations in metabolic conditions such as type 2 diabetes and non‐alcoholic fatty liver disease, may have masked potential benefits of cranberry supplementation on these metabolic markers. Another systematic review and meta‐analysis by Li et al. (2024) found significant improvements in the total cholesterol to high‐density lipoprotein cholesterol (TC/HDL‐C) ratio and insulin resistance (HOMA‐IR) with cranberry supplementation, while our study did not observe significant effects on liver enzymes (Li et al. 2024). This discrepancy may stem from differences in the metabolic pathways involved in lipid metabolism versus liver function. Cranberry polyphenols, particularly anthocyanins, are known to influence lipid metabolism and insulin sensitivity through antioxidant mechanisms and the modulation of peroxisome proliferator‐activated receptors (PPARs) and sterol regulatory element‐binding proteins (SREBPs), which likely accounts for the improvements in lipid profiles and HOMA‐IR in Li et al.'s study (Liu et al. 2025; Zhang et al. 2019). In contrast, liver enzymes are more directly impacted by factors such as hepatic inflammation and oxidative stress, which require more targeted interventions or bioactive compounds, and may not be effectively modulated by cranberry supplementation alone. Hormoznejad et al. conducted a systematic review and meta‐analysis evaluating the effects of cranberry supplementation on features of the metabolic syndrome. Their analysis, which pooled data from 10 RCTs, demonstrated a significant reduction in WC, while no significant effects were observed for lipid profiles, fasting blood glucose, or blood pressure. In contrast, our meta‐analysis did not find a significant reduction in WC (Hormoznejad et al. 2021). This discrepancy may be explained by differences in the study populations. Hormoznejad et al. focused on individuals with metabolic syndrome and type 2 diabetes, who typically have higher baseline WC and more potential for measurable reductions, whereas our meta‐analysis included a more heterogeneous population, likely attenuating the observed effect.

In the subgroup analysis of BMI, cranberry powder demonstrated a more significant effect compared to cranberry juice. This difference likely arises from the higher concentration of polyphenols in the powder, which may provide more potent metabolic benefits, while juice, with its higher sugar content and lower polyphenol levels, appears less effective (Richter et al. 2021; Xu et al. 2023). The greater BMI reduction observed in overweight individuals compared to those with obesity may be attributed to differences in metabolic responsiveness (Hirsch et al. 2016). Overweight individuals often exhibit less severe metabolic impairments, such as insulin resistance (Barazzoni et al. 2018) and inflammation (Ellulu et al. 2017), making them more receptive to interventions like cranberry polyphenols, which modulate lipid metabolism and enhance insulin sensitivity through pathways like AMP‐activated protein kinase (AMPK) and PPARγ activation (Liu et al. 2025; Xuejun Zhang et al. 2019). In contrast, individuals with obesity may require more intensive or prolonged interventions due to more resistant forms of metabolic dysfunction (Kong et al. 2025). Moreover, our subgroup analysis showed a significant reduction in BMI among individuals over 50 years of age. This may be due to age‐related metabolic dysfunction, driven by mitochondrial decline, oxidative stress, and chronic inflammation (Leyane et al. 2022). Cranberry polyphenols, particularly anthocyanins, may counteract these effects through antioxidant and anti‐inflammatory mechanisms (Caldas et al. 2018). We also observed a significant reduction in BMI following cranberry powder supplementation lasting more than 8 weeks. Notably, of the seven studies contributing to this analysis, five lasted longer than 8 weeks, and two were exactly 8 weeks; none were shorter. As a result, while these findings highlight the value of sustained cranberry supplementation (Barazzoni et al. 2018), the lack of trials under 8 weeks limits conclusions about duration‐dependent effects. Additionally, AST levels were notably higher in participants over 50, those with a BMI of 25–29.9, and individuals treated with cranberry powder for longer than 8 weeks. As AST is not liver‐specific, this elevation may reflect muscular or metabolic stress rather than hepatic injury (Thakur et al. 2024). These groups are more likely to exhibit sarcopenia (Teixeira et al. 2025; Veronese et al. 2024), insulin resistance (Arneth 2024; Zhao and Yue 2024), or early NAFLD (Ntikoudi et al. 2024), which may increase sensitivity to metabolic interventions. Prolonged exposure and the higher polyphenol content of the powder form may amplify mitochondrial and oxidative signaling, contributing to the observed increase (Richter et al. 2021; Xu et al. 2023). The absence of ALT elevation supports a non‐hepatocellular origin.

The significant reduction in BMI observed in this study highlights the potential of cranberry consumption in modulating BW. Cranberry polyphenols, particularly anthocyanins and proanthocyanidins, may reduce BMI through three primary mechanisms. First, activation of AMPK, a key regulator of energy balance, promotes lipolysis and inhibits fat storage by suppressing fatty acid synthesis and adipogenesis (Sun et al. 2016). Second, cranberry polyphenols modulate PPARγ to regulate adipogenesis (Kowalska et al. 2014). Third, their anti‐inflammatory effects reduce visceral fat accumulation by lowering inflammatory cytokines, such as TNF‐α and IL‐6 (Anhê et al. 2015). Lastly, cranberry's impact on insulin sensitivity and the gut‐liver axis further supports improved adiposity regulation (Anhê et al. 2017).

This systematic review and meta‐analysis is the first to evaluate the impact of cranberry supplementation on both anthropometric measures and liver enzyme biomarkers. Compared to previous reviews, our study incorporated larger number of RCTs across diverse populations with varying metabolic profiles, thereby enhancing the generalizability of the findings. Additionally, the use of rigorous subgroup analyses based on age, BMI, intervention type, and duration allowed for the identification of key moderators influencing cranberry's metabolic effects. However, some limitations must be acknowledged. The total number of included RCTs was relatively small, particularly for some outcomes (e.g., ALP) and subgroup comparisons, which may limit statistical power and the precision of pooled estimates. Moreover, while the pooled effect on BMI was statistically significant, the clinical relevance of a −0.30 kg/m^2^ reduction should be interpreted cautiously. Most studies had unclear or high risk of bias in at least one domain, particularly regarding allocation concealment and blinding of outcome assessment, potentially introducing performance or detection bias. Additionally, the lack of trials with intervention durations under 8 weeks restricted our ability to draw conclusions about the minimum effective exposure necessary for metabolic changes. Finally, heterogeneity in cranberry dosage, formulation (juice, extract, powder), and co‐interventions (e.g., omega‐3 supplementation or calorie restriction) introduces variability that may confound treatment effects and should be addressed in future studies using standardized protocols.

In conclusion, the current study found that cranberry supplementation significantly reduced BMI, particularly among older adults, overweight individuals, and those receiving the intervention for more than 8 weeks. While no consistent effects were observed on liver enzymes overall, AST levels increased in certain subgroups, warranting further investigation. These findings suggest that cranberry polyphenols may support weight regulation through metabolic and anti‐inflammatory pathways, though their effects on liver function remain uncertain. Future RCTs should directly compare different forms of cranberry consumption, including conventional food sources (fresh or dried fruit, tea infusions, and juice) and dietary supplements standardized for bioactive compounds, in order to clarify differences in efficacy, bioavailability, and metabolic safety.

## Author Contributions

**Farhang Djafari:** writing – original draft, methodology. **Mohammadreza Hosseinpour Dogolsar:** writing – review and editing, writing – original draft. **Mohammad Reza Amini:** writing – original draft, formal analysis, methodology, conceptualization. **Kimia Fathzadeh:** writing – original draft. **Shadi Ghaemi:** writing – original draft. **Sogand Tavakoli:** writing – original draft. **Fatemeh Sheikhhossein:** writing – original draft. **Azita Hekmatdoost:** writing – original draft, writing – review and editing, supervision.

## Funding

This study is related to the project NO. 1403/60174 From Student Research Committee, Shahid Beheshti University of Medical Sciences, Tehran, Iran. We also appreciate the “Student Research Committee” and “Research & Technology Chancellor” in Shahid Beheshti University of Medical Sciences for their financial support of this study.

## Conflicts of Interest

The authors declare no conflicts of interest.

## Data Availability

The data used to support the findings of this study are available from the corresponding author upon request.
